# MicroRNAs and Transcripts Associated with an Early Ripening Mutant of Pomelo (*Citrus grandis* Osbeck)

**DOI:** 10.3390/ijms22179348

**Published:** 2021-08-28

**Authors:** Heli Pan, Shiheng Lyu, Yanqiong Chen, Shirong Xu, Jianwen Ye, Guixin Chen, Shaohua Wu, Xiaoting Li, Jianjun Chen, Dongming Pan

**Affiliations:** 1 College of Horticulture, Fujian Agriculture and Forestry University, Fuzhou 350002, China; panheli@fafu.edu.cn (H.P.); lsh0809@zafu.edu.cn (S.L.); 2840@mju.edu.cn (Y.C.); 1200305016@fafu.edu.cn (S.X.); 1170371005@fafu.edu.cn (G.C.); 000q020040@fafu.edu.cn (S.W.); 2170305003@fafu.edu.cn (X.L.); 2 Department of Environmental Horticulture and Mid-Florida Research and Education Center, Institute of Food and Agricultural Sciences, University of Florida, Apopka, FL 32703, USA; 3 State Key Laboratory of Subtropical Silviculture, Zhejiang Agricultural and Forestry University, Hangzhou 311300, China; 4 Institute of Oceanography, Minjiang University, Fuzhou 350108, China; 5 Agricultural and Rural Bureau of Pinghe County, Zhangzhou 363700, China; 1200305006@fafu.edu.cn

**Keywords:** *Citrus grandis*, microRNAs, fruit ripening, pomelo

## Abstract

‘Liuyuezaoyou’ is an early-ripening cultivar selected from a bud mutation of *Citrus grandis* Osbeck ‘Guanximiyou’. They were designated here as MT and WT, respectively. The fruit of MT matures about 45 days earlier than WT, which was accompanied by significant changes in key phytohormones, sugar compounds and organic acids. Recent studies have showed that microRNAs (miRNAs) play an important role in regulation of fruit ripening process. The aim of this study was to compare MT fruits with WT ones to uncover if miRNAs were implicated in the ripening of *C. grandis*. Fruits of both WT and MT at four developmental stages were analyzed using high-throughput sequencing and RT-PCR. Several independent miRNA libraries were constructed and sequenced. A total of 747 known miRNAs were identified and 99 novel miRNAs were predicted across all libraries. The novel miRNAs were found to have hairpin structures and possess star sequences. These results showed that transcriptome and miRNAs are substantially involved in a complex and comprehensive network in regulation of fruit ripening of this species. Further analysis of the network model revealed intricate interactions of miRNAs with mRNAs during the fleshy fruit ripening process. Several identified miRNAs have potential targets. These include auxin-responsive protein IAA9, sucrose synthase 3, V-type proton ATPase, NCED1 (ABA biosynthesis) and PL1/5 (pectate lyase genes), as well as NAC100 putative coordinated regulation networks, whose interactions with respective miRNAs may contribute significantly to fruit ripening of *C. grandis*.

## 1. Introduction

Fleshy fruits have evolved attractive color, taste, and aroma to recruit animal vectors for seed dispersal. Today, fleshy fruits are integral components of nutrition for the human diet. Based on the respiration rate of fruit ripening, fleshy fruit can be classified into two groups: climacteric and non-climacteric fruits [1]. Climacteric fruits display a dramatic change in respiration, together with autocatalytic ethylene synthesis at the onset of fruit ripening. The ripening mechanism of climacteric fruits has been well established and genes or enzymes committed to these metabolic steps have been discovered [2]. Climacteric fruit ripening and senescence also have been shown to operate at the transcriptional, posttranscriptional, and translational levels [3,4]. Furthermore, microRNAs (miRNAs) have been identified to involve in the climacteric fruit ripening process by deep sequencing [5,6,7,8]. Small RNA/miRNAs are universal and highly conserved 20–24 nucleotides (nt) long, noncoding RNAs that have been shown to be major regulators of gene expression in eukaryotic organisms. Ripening mutants and transgenic methods have been employed to explore the functions of these miRNAs as negative regulators of their target genes during ripening in different tomato varieties [9].

Non-climacteric fruits, such as citrus, cherry, grape, litchi, pear, and strawberry, exhibit a rather constant respiration rate and produce little or no ethylene. Unlike climacteric fruits that are usually harvested before their maturity, non-climacteric fruits need to stay on plants in order to reach full physiological maturity. Once harvested, they will not continue to mature and not gain any flavor or sugar. Citrus is one of the most commonly consumed non-climacteric fruits in terms of health-promoting plant secondary metabolites. Fruits with different ripening times have different quality, storage tolerance and disease resistance. The maturity and harvest time of citrus fruits influences fruit storage, preservation, commodity management, transport, and marketing work [10,11]. Citrus fruit ripening is a complex process that involves dramatic changes in various regulatory events, metabolic reactions, and physiological and biochemical processes. These changes strongly affect their shelf life and economic value, such as fruit color, taste, sugar, acid, aroma, texture, and nutritional value associated metabolisms [3,12,13,14]. Thus, elucidating citrus fruit ripening regulatory pathways and networks is important for the improvement of citrus cultivars. However, evidence is still limited about miRNAs in regulation of development and ripening of non-climacteric fruits, such as citrus.

Pomelo or pummelo (*Citrus grandis* Osbeck) is the largest citrus fruit in the family Rutaceae and regarded as the ancestor of grapefruit. It is a naturally occurring, non-hybrid citrus fruit and native to Southeast Asia. Zhangzhou, located in southern Fujian Province, China has a long history in pomelo production and is particularly rich in pomelo landraces. ‘Guanximiyou’ is one of the most popular cultivars producing large fruit, about one kilogram each. The juice sac of this cultivar contains high vitamin C, averaging 34 mg/100 g. In 2007, a bud mutant was identified from ‘Guanximiyou’, which has the same characteristics as its parental plants but differs in fruit maturity. The fruit of the mutant matures 45 days earlier than ‘Guanximiyou’.

This study was intended to investigate the fruit ripening of pomelo using the mutant and its parental plant as materials. ‘Guanximiyou’ designated as wild type (WT) and ‘Liuyuezaoyou’ as mutant (MT) were analyzed to specifically address if miRNA-mediated gene silencing could play a role in the transcriptional network of pomelo fruit ripening. RNA sequencing and miRNA expression profiling using high-throughput sequencing were performed for the two cultivars. Differential expression analysis and gene set enrichment were conducted to examine the role of citrus miRNAs in modulating fruit ripening process. It was anticipated that a better understanding of the underlying mechanisms in fruit ripening would accelerate genetic improvement of pomelo through biotechnological means.

## 2. Results

### 2.1. Physiological and Metabolic Differences between WT and MT

**Figure 1:**
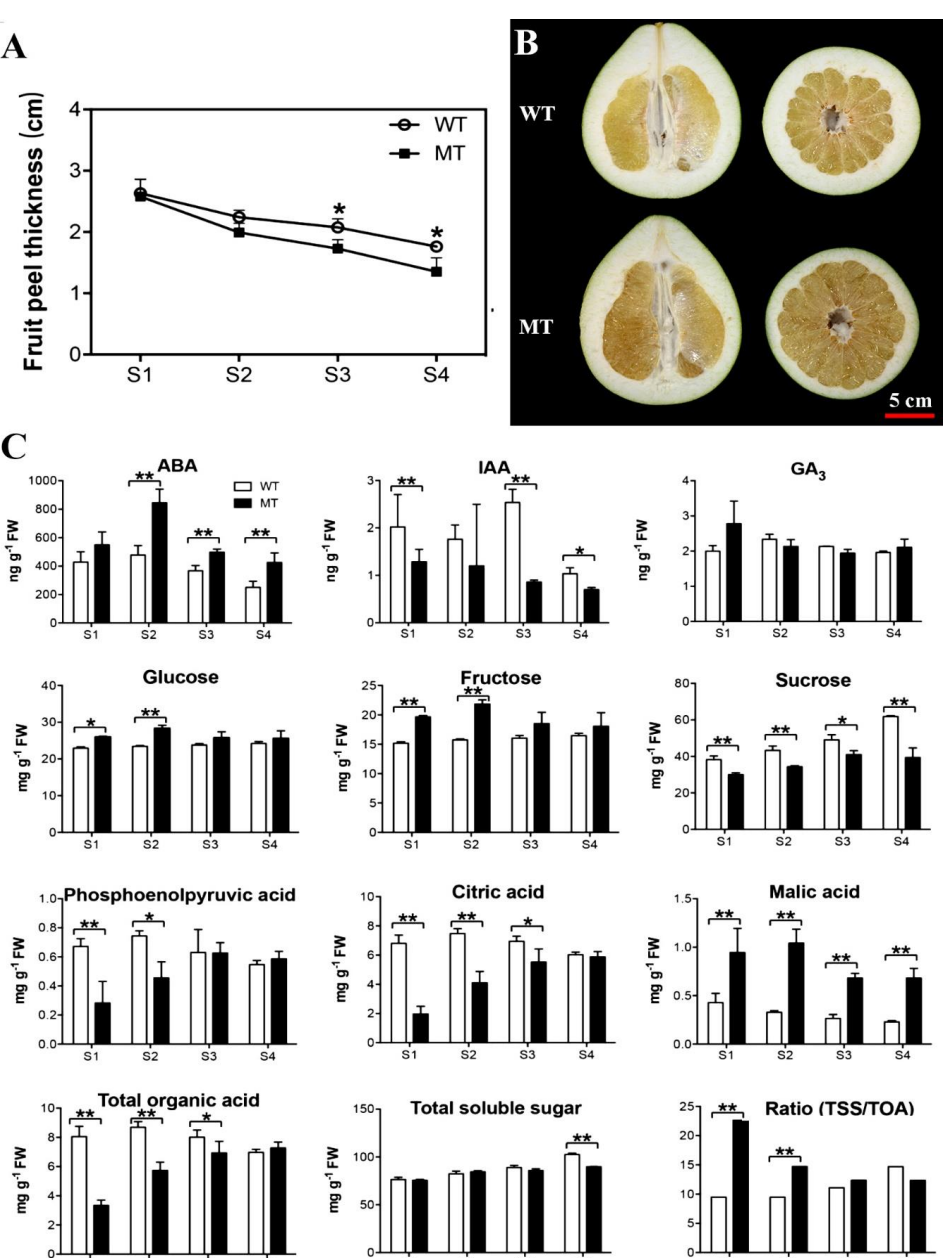
Morphological changes in two *Citrus grandis* cultivars during juice sac development. (**A**) Fruit peel thickness of WT and MT during four ripening stages. (**B**) The cross sections of peel thickness between WT and MT at ripening stage 4. (**C**) Content of phytohormones, sugar and organic acids of WT and MT fruits during the four ripening stages: S1, S2, S3 and S4 indicate 76, 90, 104 and 118 days after flowering, respectively. * and ** indicate significant differences at *p* < 0.05 and *p* < 0.01 levels, respectively between WT and MT at a particular ripening stage based on Student’s *t*-test.

The fruit peel thickness of WT and MT was similar at stage one of fruit ripening (S1), i.e., 76 days after flowering (DAF) (Figure 1A). The peel thickness of MT started to become thinner than WT at S2 (90 DAF). From S3 (104 DAF), which is known as commercially mature to S4 (118 DAF) as fully mature, the peel thickness of MT fruit was significantly thinner than that of WT (Figure 1A). In pomelo ripening, the peel thickness generally decreased with the juice sac expansion. The cross sections of WT and MT fruit in Figure 1B show the peel thickness at S4. Morphologically, S3 appeared to be the turning point of fruit ripening in MT as the fruit peel thickness significantly decreased and peel hardness started softening compared to those of WT.

Fruit biochemical parameters varied greatly between WT and MT during the ripening process. As shown in Figure 1C, different endogenous hormones exhibited variable trends during the process of maturation. Abscisic acid (ABA) content in MT was significantly different from WT from S2 to S4. ABA content in MT sharply increased at S2 and then decreased to 400 ng/g FW at S4, indicating that ABA in MT underwent a process of rapid accumulation and degradation. While ABA levels in WT showed a slight increase at S2 and slow decrease until S4. The indole-3-acetic acid (IAA) content fluctuated in WT. There was a significant increase at S1, slight decrease at S2, a dramatic increase at S3 and then decrease at S4. The IAA contents in MT showed an overall downward trend and were significantly lower than WT at S1, S3 and S4. Gibberellic acid (GA_3_) contents were rather stable during the ripening process in both WT and MT. ABA is the most important hormone in regulating ripening of non-climacteric fruits. The peak of ABA concentration in MT at S2 could indicate the initiation of fruit ripening, which subsequently led to the reduced fruit peel thickness at S3 and S4. On the other hand, IAA generally plays opposite roles to ABA in regulation of fruit ripening. Thus, IAA levels in WT were significantly higher at S1, S3 and S4 compared to MT. The significant differences between WT and MT in ABA and IAA contents could implicate the two hormones in regulation of the maturity of pomelo fruit.

The type, content, and ratio of sugar to organic acid in pomelo pulp determine the flavor of the fruit and are also important indicators for measuring the maturity. Thus, the main soluble sugars (glucose, fructose, and sucrose) as well as phosphoenolpyruvic acid, citric acid and malic acid in both WT and MT were analyzed by LC-MS/MS. The contents of soluble sugars and organic acids in WT and MT differed greatly during the ripening process (Figure 1C). Among them, the main soluble sugars were glucose, fructose, and sucrose. Glucose and fructose concentrations in MT increased from S1 to S2. The levels of glucose and fructose in MT were higher than those in WT at S1 and S2, while the content of sucrose in WT was significantly higher from S1 to S4 than that of MT. It was worthy to note that glucose, fructose, and sucrose levels in MT at S3 were almost the same as those at S4, indicating that with the decrease of fruit peel thickness from S3, sugar compounds become stabilized.

The main organic acids in pomelo fruit were citric acid, phosphoenolpyruvate, and malic acid. In general, there was an increase in phosphoenolpyruvate, citrus acid, and total organic acid in MT from S1 to S3 and they became plateaued at S4. Phosphoenolpyruvate contents in WT were significantly higher than MT at S1 and S2 and became comparable at S3 and S4. The citric acid contents in WT were significantly greater than MT from S1 to S3. There was a decrease in malic acid contents in both WT and MT from S1 to S4. Interestingly, the malic acid content in MT was significantly higher than that of WT throughout the ripening process. Overall, total organic acids were higher in WT than MT, total soluble sugar was similar between the two from S1 to S3 but significantly higher in MT at S4. As a result, the ratio of total soluble sugar to total organic acids at S1 and S2 were much greater in MT than WT and become comparable at S3 and S4. These results indicated that the early fruit maturity of MT was accompanied by dynamic changes in organic acids and the ratio of sugar to acid compounds.

### 2.2. An Overview of High-Throughput Sequencing

To determine if the morphological and biochemical variation in fruit maturity between WT and MT was related to different mRNA and miRNA abundance, we constructed mRNA and small RNA (sRNA) libraries using the WT and MT juice sacs collected from the four fruit developmental stages. A total of 129,758,793,600 raw bases and 648,793,968 unique reads were generated from the 24 libraries (Table 1). After removing the adapter sequences, low quality reads and short reads, the unique sRNAs were annotated using the Rfam database to exclude small nucleolar RNA (snoRNA), small nuclear RNA (snRNA) and ribosomal RNA (rRNA) sequences. To detect known miRNAs, clean reads were mapped to the *Citrus grandis* reference genome sequence (http://citrus.hzau.edu.cn/orange/ accessed on 24 August 2021) and miRbase and then miRDeep2 was used for novel miRNA prediction. A total of 747 known miRNAs were identified and 99 novel miRNAs were predicted across all libraries. Novel miRNAs were all found to have hairpin structures and possess star sequences (Table 2).

**Figure 2:**
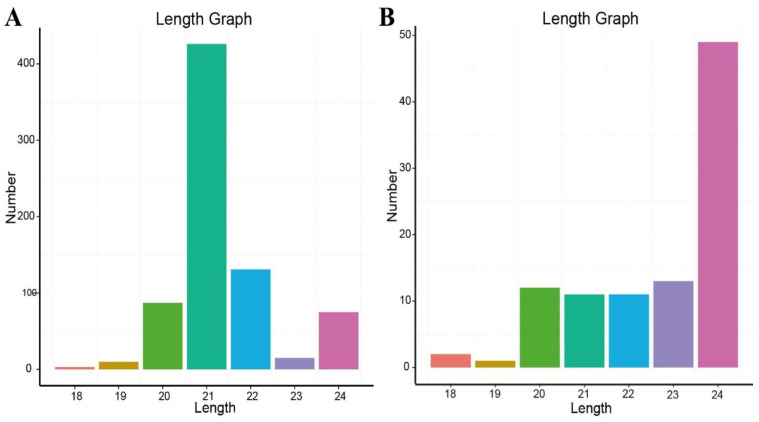
The length distribution of conserved miRNAs (**A**) and novel miRNAs (**B**).

In order to characterize the small RNAs, the length distributions of the miRNAs (known and novel) were analyzed and plotted (Figure 2). Among these conserved miRNAs, the most abundant reads were found to be 21 nt in length (Figure 2A) and the most abundant novel miRNAs had a sequence of 24 nt (Figure 2B). The dominance of the 24 nt length sequences suggested that they were small interfering RNA (siRNA) [15], which is consistent with previous reports from other species, including *Arabidopsis thaliana* [16], *Oryza sativa* [17], *Solanum lycopersicum* [18], *Zea mays* [19], sorghum [20], *Prunus avium* L. [21] and *Capsicum chinense* [22].

### 2.3. Comparative Analysis of miRNAs and Their Expression Profiles in WT and MT Juice Sacs during Fruit Ripening

**Figure 3:**
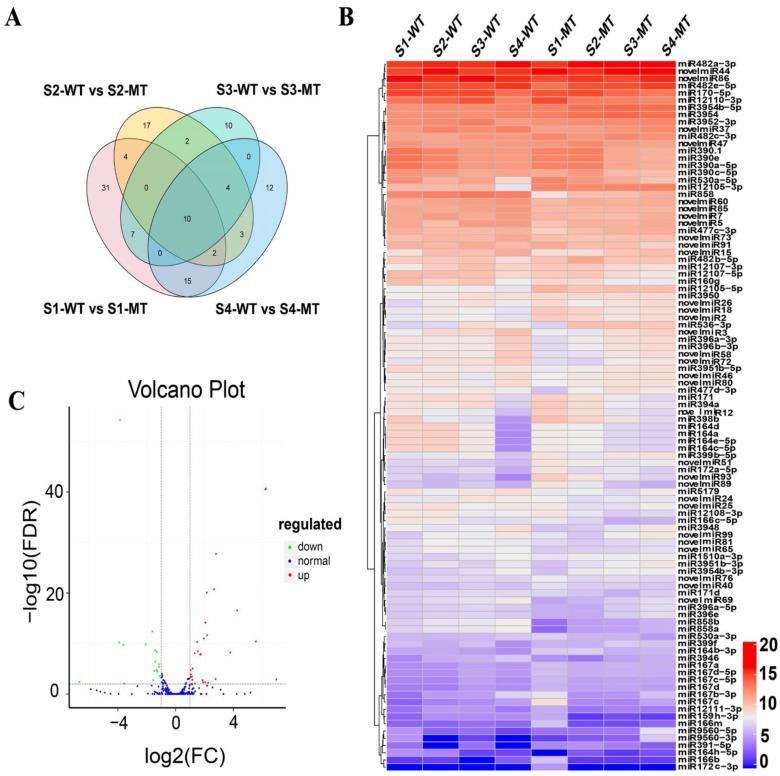
Differentially expressed miRNAs in fruit sacs of WT and MT. (**A**) Venn diagrams showing the number of the differentially expressed miRNAs in the four developmental stages of fruit ripening. (**B**) Heatmap showing the expression pattern of the differentially upregulated and downregulated miRNAs in fruit of WT and MT during the four developmental stages. The clusters were generated based on the Pearson correlation coefficient of normalized miRNA expression. (**C**) Volcanic diagrams showing the number of differentially expressed miRNAs between WT and MT at S3. Where the red dots indicate up-regulated miRNAs with significant differences, the green dots indicate down-regulated miRNAs with significant differences and the blue dots indicate those that were not significant for miRNA expression. FC = fold change; FDR = false discovery rate.

The Venn diagrams (Figure 3A) shows that the 10 differentially expressed (DE) miRNAs were presented in all groups, meaning that the 10 miRNAs were exclusively involved in all stages of fruit ripening. On the other hand, 31, 17, 10 and 12 miRNAs were only implicated in S1, S2, S3 and S4 of fruit ripening, respectively.

Several known miRNA family members were identified in all developing stages (Figure 3B), including miR482a-3p, miR482e-5p and miR170-5p, which were highly differentially expressed in both WT and MT. Although some miRNAs were from the same family, each family had multiple members, such as miR482. The expression level of miR482a-3p increased sharply in MT after S2 but fluctuated in WT. On the other hand, miR482e-5p expression showed an increasing trend from S1 to S4 in both WT and MT. The abundance of miR170-5p increased during the early fruit ripening in both WT and MT but decreased in the late ripening stages, while miR12110-3p expression was higher at S1 but became downregulated from S2 to S4 in MT and its expression in WT showed an increase from S1 to S3 followed by a decrease at S4.

Novel miRNAs, such as miRn44, miRn86, miRn37 and miRn47, were highly abundant in all libraries. The expression of miRn37 fluctuated in WT but exhibited an increasing trend in MT. The level of miRn47 was higher in WT at S1 then decreased thereafter; but its expression in MT was lower at S1, increased at S2 and then decreased. The expression of other novel miRNAs appeared to be variable in WT and MT at different developmental stages of fruits.

A volcanic diagram (Figure 3C) is presented to showed that the differentially expressed miRNAs in WT and MT at S3, which showed more than 25 miRNAs were upregulated and 15 miRNAs were downregulated at S3.

### 2.4. Analysis of Differences in Transcriptions between WT and MT Fruits

**Figure 4:**
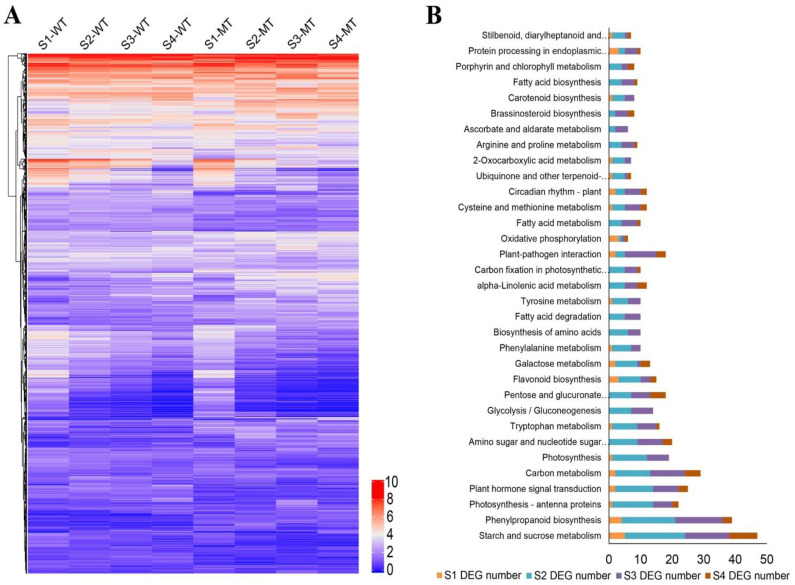
Differentially expressed mRNAs in WT and MT. (**A**) Heatmap showing the expression pattern of differentially expressed genes (DEGs) in the fruits of WT and MT. (**B**) The enrichment of DEGs in top 20 KEGG pathways, where orange, light green, purple and dark red bars indicate the number of DEGs at S1, S2, S3 and S4 stages, respectively.

Based on differentially expressed genes (DEGs) in WT and MT, all DEGs were clustered into 10 groups, presented as heatmaps in Figure 4A. Numerous miRNAs and their target genes were identified according to sequence homology, which were known to be potentially involved in the fruit ripening process. These included genes in the ethylene biosynthesis and ethylene signaling and response pathways: ethylene-responsive transcription factor; ABA biosynthesis and signal transduction pathways: NCED1 (9-cis-epoxycarotenoid dioxygenase); IAA biosynthesis and signal transduction: auxin-responsive protein IAA9. In addition, differential expression mRNA encoding several key genes involved in cell wall metabolism, carbohydrate transport and metabolism during fruit development, such as pectate lyase 1/5, xyloglucan endotransglucosylase, expansin-A1, pectin acetylesterase 8, beta-glucosidase 3/11, sucrose synthase 3 and cell wall inhibitor of fructosidase 1.

KEGG pathway enrichment analysis was carried out based on DEGs. Figure 4B shows that there were at least four DEGs were enriched in each of the 30 KEGG metabolic pathways during the course of fruit ripening, of which more enrichment occurred in the middle of fruit developmental stages, i.e., S2 and S3. Among them, five metabolic pathways including starch and sucrose metabolism, phenylpropanoid biosynthesis, photosynthesis-antenna proteins, plant hormone signal transduction and carbon metabolism had the most DEGs. Moreover, 22 DEGs were enriched in the plant hormone signal transduction pathway. Carotenoid biosynthesis, tryptophan metabolism and brassinosteroid (BR) biosynthesis are the metabolic pathways related to endogenous hormones ABA, IAA, and BR biosyntheses. The KEGG pathway results highlighted differences in gene expression in plant hormone signaling and starch and sucrose metabolism, indicating their independent or joint actions in the fruit ripening process.

### 2.5. Target Analysis of miRNA in Fruit

**Figure 5:**
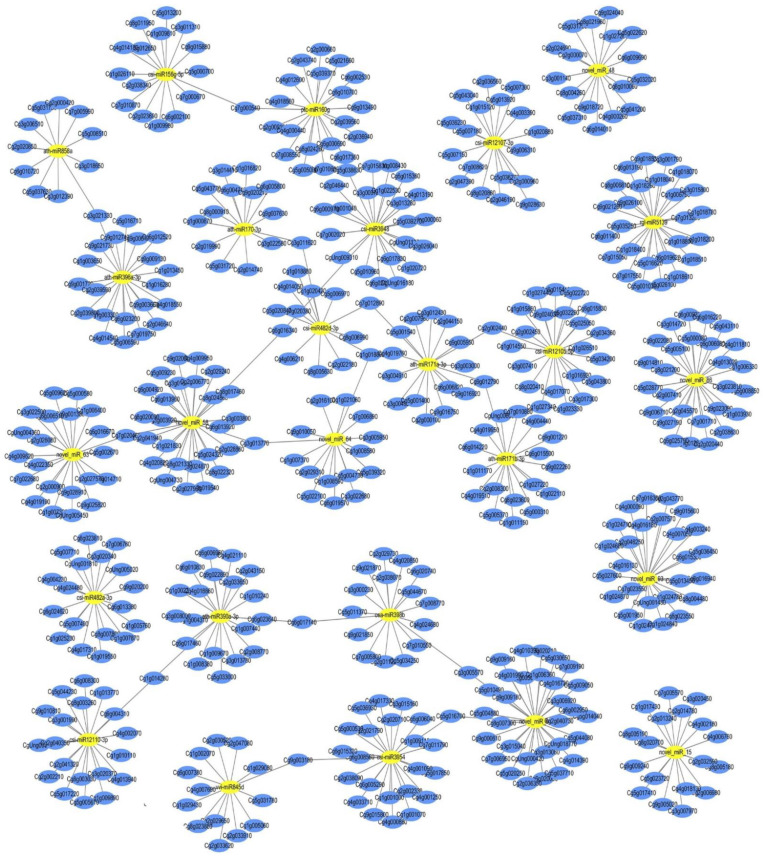
Target genes analysis of miRNAs. Relationships between miRNAs and their targets. Yellow circles represent the differentially expressed miRNAs and blue circles represent the target genes of differentially expressed miRNAs.

To further investigate the potential roles of the differentially expressed miRNAs and miRNA species in regulation of fruit development, target genes were predicted using the psRNATarget program (Additional file 1: Table S1). Based on the assignment of the functional category annotations, various relationships between miRNAs and their target genes were outlined (Figure 5).

Four miRNAs, including csi-miR9560-5p targeting auxin transporter-like protein 1, miR165a-3p targeting auxin-responsive protein IAA9, miR7712-5p targeting IAA-amino acid hydrolase and miR172c targeting indole-3-acetic acid-induced protein were found in regulation of auxin signaling as well as auxin perception. Furthermore, csi-miR391-5p was predicted to target abscisic stress-ripening protein and csi-miR12106-3p was found to target BES1/BZR1 (brassinosteroid insensitive 1 EMS-suppressor1 and brassinazole resistant 1) homolog protein 2. The two target genes were associated with ABA and BR signaling pathways, respectively. ERF023 (ethylene-responsive transcription factor) and AP2/ERF and B3 domain-containing transcription repressor were predicted to be modulated by novel miRn63 and miR159a-5p. MADS-box proteins regulating fruit-ripening were shown to be regulated by novel miRn32 and miR827-3p. WUSCHEL-related homeobox 4 was the possible target genes of csi-miR477c-3p. WRKY transcription factor 53/41 was found to serve as the target genes of csi-miR857/miR171a-3p. NAC domain-containing protein 100 was predicted to act as the target genes of miR64a. Several DEGs in cell wall and carbohydrate metabolism including UDP-glycosyltransferase, xyloglucan endotransglucosylase, β-galactosidase, endoglucanase, sucrose synthase were predicted targets of miRNAs as well. Differentially expressed miRNAs also could target different genes related to fruit ripening. For example, miRn64 was the regulator of auxin-responsive protein IAA9, alpha-trehalose-phosphate synthase and polygalacturonase. Additionally, miR160g was predicted to degrade auxin response factor 6 and glucan endo-1,3-beta-glucosidase 4. Endoglucanase 6 and WRKY41 were the potential targets of miR171a-3p.

To better understand the roles of miRNAs in the transcriptional regulation of fruit development, we also analyzed miRNAs targets for COG (clusters of orthologous groups) enrichment annotations. COG enrichment analysis revealed that signal transduction mechanisms, amino acid transport and metabolism, carbohydrate transport and metabolism, cell wall/membrane/envelope biogenesis and secondary metabolites biosynthesis, transport and catabolism were among the significantly enriched processes (Additional file 2: Figure S1).

**Figure 6:**
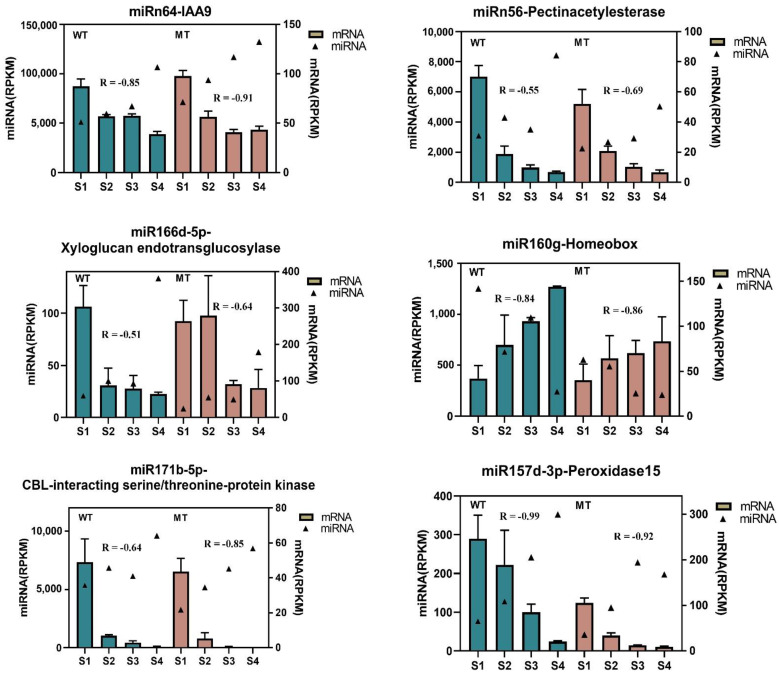
Selected results on expression correlation analysis between miRNAs and their targets during the four stages of pomelo fruit ripening of WT (dark green bars) and MT (brown bars). Triangles represent the expression level of miRNA, bars represent the expression level of targeted mRNA. The relationship between the miRNA and its targeted mRNA in WT and MT was analyzed, respectively based on Pearson’s correlation analysis and R represents correlation coefficients.

In general, miRNAs negatively regulate the expression of their targets by either degradation or posttranscriptional inhibition. There were high levels of negative correlations between miRNAs and target genes during pomelo fruit ripening. Figure 6 shows opposite expression patterns of six pairs of miRNA-target. For example, miRn64 targeted auxin-responsive protein IAA9, which was involved in regulating auxin signaling as well as auxin perception. The increased expression of miRn64 resulted in the reduced expression of IAA9 from S1 to S4 in both WT and MT. The expression level of target genes (pectin acetylesterase and xyloglucan endotransglucosylase) also displayed the opposite trend, which were involved in cell wall metabolism. The conserved miRNAs (miR157d-3p and miR171b-5p) showed significant increase in expression, whereas the target genes peroxidase 15 and CBL-interacting serine/therionin kinase exhibited decreased responses. On the other hand, there were decreased expression of miR160g, which resulted increased expression of transcript factor homeobox from S1 to S4.

### 2.6. Verification of Differentially Expressed miRNAs and Target Genes

**Figure 7:**
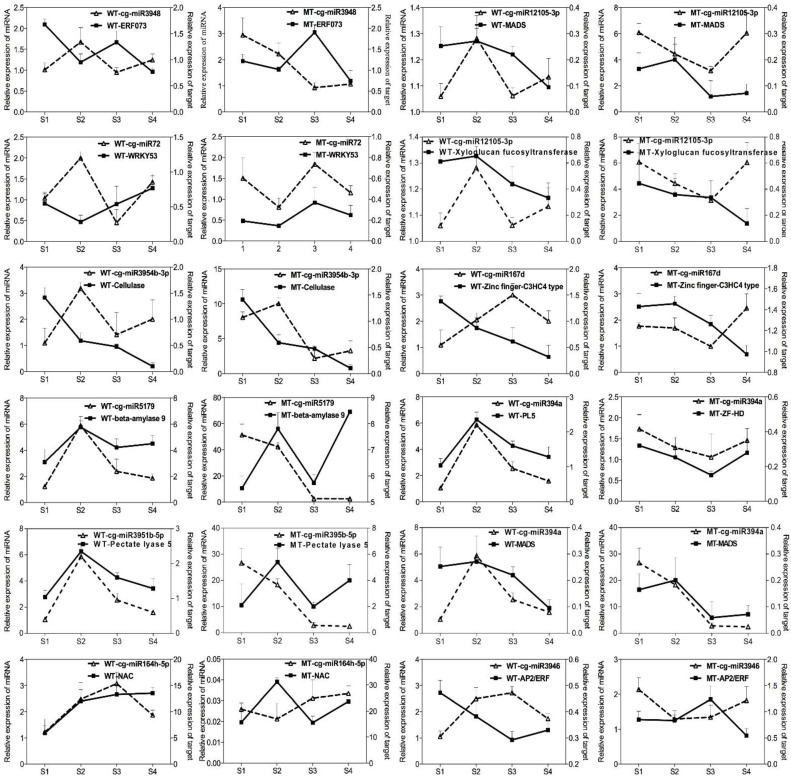
Quantitative real-time PCR validation of 12 differentially expressed miRNA and their target genes at different fruit ripening stages in WT and MT juice sacs. The data are presented as the mean ± S.D. of three independent experiments.

To validate the regulatory function of the miRNAs, a novel miRNA and 11 conserved miRNAs were analyzed by RT-qPCR. Figure 7 shows the expression profiles of 12 miRNAs and their target genes during the ripening of WT and MT. Among them, cellulase, pectate lyase 5, beta-amylase and xyloglucan endotransglucosylase showed similar expression patterns between MT and WT; however, the expression levels of these genes were significantly higher in MT due to the negative regulation of corresponding miRNAs. These target genes were involved in fruit softening and the increased upregulation of these genes in MT could accelerate ripening of MT.

Predicted targeted differential expressed miRNAs were used to elucidate the relationship between functions and phenotypes. The expression profiles of *WRKY53*, *MADS*, *NAC*, *AP2/ERF*, *zinc-finger-C3H4* and *ZF-HD* target genes were analyzed, respectively (Figure 7). The miRNA abundances of cg-miR3948 and target gene ERF073 in WT fluctuated during the ripening, but the expression pattern was opposite between cg-miR3948 and the target in MT. The expression of *WRKY53* in WT fruits decreased during the S1 to S2 stages and then increased towards the end of ripening. miRn72 was negatively correlated with *WRKY53* in WT but positively in MT fruit. This phenomenon was also observed in other miRNA-targets groups. *NAC* was predicted as the target of miR164h-5p, in MT, the expression of cis-miR164h-5p was negatively correlated with *NAC* gene. However, in WT, this reverse expression pattern only appeared at the end of development, which indicated the target genes were regulated by miRNAs in MT and WT. The expression of miR3946 increased with the ripening of the WT fruit and reached the peak at S3 stage when fruit was fully ripened, this was followed by a rapid decrease toward S4 stage. The expression pattern of *AP/ERF* indicated that miR3946 severely repressed the *AP/ERF* expression. Moreover, the expression of miR3946 and *AP/ERF* in MT plant showed different trends of change in WT, but negative correlation occurred between them in MT fruit. 

## 3. Discussion

This study analyzed temporal changes in some phytohormones, sugar compounds and organic acids in an early fruit maturing mutant of pomelo and its wild type and investigated miRNA involvement in the process of fruit ripening. Through a systematic comparison, our results showed that the early maturity was associated with dynamic changes in the content of ABA, IAA, sugar compounds and organic acids and miRNAs were heavily involved in the early ripening process. This study for the first time documented 747 known miRNAs and 99 novel miRNAs that are implicated in fruit ripening of pomelo. The targets of some miRNAs were further analyzed, and results showed that miRNA targets affect primary metabolism, cell-wall degradation, phytohormone and transcription factors. Such coordinate interactions may substantially contribute to pomelo fruit development and its ripening.

### 3.1. MiRNA Targets Affect Primary Metabolism

Ripening of non-climacteric fruits is accompanied by the changes in both primary and secondary metabolism [23], during which carotenoids, sugars, anthocyanidin and other soluble compounds are accumulated, while organic acid and chlorophyll contents are reduced [24,25,26]. In this study, sugar and organic acid concentrations in juice sacs were significantly altered. Several miRNAs, such as miR3627-5p, csi-miR482d-5p, csi-miR530a-5p and miR9480a were identified and they could potentially target sugar- or organic acid-metabolic pathways. For example, sucrose synthase 3 was the potential target of csi-miR482d-5p, which was highly expressed in early ripening fruit juice sacs. This is coordinated with the increased content of sucrose from S1 to S4 (Figure 1C). Sucrose can also act as a signal involved in non-climacteric fruit development and ripening [27]. Six sucrose synthase (Sus) genes were identified in citrus genome. Among them, *CitSus1, 2, 5* and *6* were predominantly expressed in fruit juice sacs. During fruit development, *CitSus5* transcript increased and *CitSus6* transcript decreased significantly in fruit [28]. In addition, sugar transporters were also related to fruit senescence, the sugar accumulation pattern, or other essential fruit biological processes [29,30]. In MT fruit, the expression level of sugar transporter ERD6-like 16 was significantly higher than WT from S2 and S3 and the non-conserved miR73 is likely the regulator of sugar transporter ERD6-like 16. Organic acids play a key role in fruit development, maturation, and ripening [31]. High-throughput sequencing showed that ATPase 10 and V-type proton ATPase were highly expressed in MT at S1, which could be regulated by miR6483 and csi-miR530a-5p, respectively. ATPase is related to citrate storage into the vacuole [32].

### 3.2. MiRNA Targets Regulate Cell-Wall Degradation

Fruit ripening is associated with changes in cell wall fractions, such as pectin [33,34]. A large number of pectin degrading enzymes have been characterized from plants and they are implicated in numerous aspects of fruit development [35]. The action of pectate lyase results in plant cell-wall degradation through the release of oligogalacturonides from the plant cell wall [34]. Silencing the expression of pectate lyase genes in strawberry and tomato resulted in prolonged fruit firmness [36,37]. In the present study, two pectate lyase genes (*PL1/5*) were regulated by miR399b and csi-miR3951b-5p, which resulted in a high mRNA expression level in MT juice sacs. Pectinesterase (PE) could serve to either strengthen or weaken the cell wall depending on its mode of action and the environment. Its silencing resulted in increased rate of fruit softening during ripening [38]. RNA-Seq results showed that PE 40 greatly decreased in early ripening fruits. The degree of acetylation of pectin can be modulated by pectin acetylesterase, which plays diverse roles in plant development [39]. In the present work, we found that the transcript level of pectin acetylesterase 8 increased during fruit maturity. Furthermore, pectin acetylesterase 8 was highly expressed in MT compared with WT at S2 and S3. These actions could contribute to the accelerated ripening in MT.

Xyloglucan endotransglucosylase (XTH) plays a dual role in integration of newly secreted xyloglucan chains into an existing wall-bound xyloglucan or restructuring the existing cell wall material by catalyzing transglucosylation between previously wall-bound xyloglucan molecules, which results in the maintenance of the structural integrity of the cell wall involved in tomato fruit softening [40,41]. In the current study, mRNA for xyloglucan endotransglucosylase was highly abundant in MT fruit in the early developmental stages and xyloglucan endotransglucosylase was predicted as the target of miR9480a. A discrete group of *FvXTHs* increased their expression during softening of wild strawberry fruit, which was required for cell wall remodeling in fruit softening [42]. Expansin-A1 was up-regulated in MT fruit from S2 to S4, it is the target of miR8175 (Table 2). Expansins are essential for cell enlargement and cell wall loosening during many developmental processes in plants [43]. Thus, the upregulation of expansin-A1 could be implicated in fruit softening.

### 3.3. MiRNA Targets Phytohormone Related Genes

Phytohormones are essential for fruit development and maturation [44]. Several lines of evidence suggest that ABA plays an important role in inducing fruit maturity in non-climacteric type [45,46]. *NCED* genes are critical to ABA biosynthesis, which is regulated by internal signals or external environment [47]. A noticeable difference between WT and MT was the higher ABA content in MT from S2 to S4. Results showed that *NCED1* triggered by miR164a showed a high expression level in MT at S2.

Other hormones, such as IAA and GA are also important in fruit ripening. The levels of IAA and GA_1_ rise early in strawberry development then drop to low levels prior to color accumulation, the opposite pattern was observed for ABA [48]. Previous reports showed that miRNA induction was involved in regulating auxin signaling via miR160, miR167 and miR393. The transcription levels of auxin-responsive protein IAA9 decreased continuously during the ripening process. Meanwhile, the expression levels of miRn64 in MT and WT were sharply increased and reached a peak in S4 when fruit was fully ripened (Figure 7). Other auxin or GA responsive target genes, such as IAA-induced protein, auxin transporter, gibberellin-regulated protein 1 and gibberellin receptor GID1B also showed different expression levels between WT and MT. Among them, miR390 triggers TAS3 to produce ARF-tasiRNAs, which targets ARF TFs that is involved in organ developmental timing and patterning [49]. In tomato, *SlARF7/SlIAA9* and *SlDELLA* antagonistically modulate the expression of feedback-regulated genes involved in GA and auxin metabolism, *SlARF7/SlIAA9* and *SlDELLA* coregulate the expression of fruit growth-related genes to prevents GA biosynthesis and auxin metabolism via inhibiting *GA20ox1/GA3ox1* and *GH3.2* expression [50,51].

Fruit development is fine-tuned by multiple ripening transcription factors (TFs) ethylene signaling components, such as ETHYLENE RESPONSE FACTOR (ERF). Four different ERF TFs (*ERF RAP2-3*, *ERF012*, *ERF023* and *AP2/ERF-B3* domain-containing transcription repressor) were found to be differentially expressed between WT and MT during fruit ripening. Expression levels *ERF1* and *ERF2* were repressed by ethylene, which supports the involvement of ethylene in the regulation of some aspects of peel maturation in the citrus fruit [52]. In the climacteric fruit, ERFs can also act as key genes in ethylene signaling pathway which directly regulated downstream genes to control hormone synthesis, carotenoid synthesis and fruit softening (*ACO*, *ACS*, *EXP*, *PSY*, *XET*, *PME* and *PG*) [53,54,55,56,57]. The comparative analysis of DE miRNAs between the WT and MT fruits suggested that their specific changes are involved in ethylene pathways, such as miR172a (target AP2 ethylene-responsive transcription factor-TOE3), miR396a-5p and miR477-3p (target ETHYLENE INSENSITIVE 3-like), miR9470-3p (ERF5 and ERF021) [4]. The non-conserved tomato slymiR1917 mediates degradation of *SlCTR4* splice variants (*SlCTR4sv*) regulate ethylene responses in tomato [9,58].

### 3.4. MiRNA Targets TFs Related to Fruit Ripening

Transcription factors are key regulators in control of gene expression [59]. RT-qPCR analysis showed relative expression of the key TFs in juice sac development (Figure 7). A large number of TFs are implicated in fruit development and metabolism, such as TFs belonging to the MYB [60], NAC [61], bHLH [62] and MADS [63] families. Moreover, many of the target genes of miRNAs and tasiRNAs are TFs [64]. For example, miR156 was predicted to regulate CNR (colorless non-ripening, SPL TF) to control ripening in tomato [65], SPL TFs are also related to phase change and trichome development in *Arabidopsis* and perennial trees, miR164 mediates NAC TF [66]. Furthermore, in *Arabidopsis*, rice and kiwifruit, flower development is critically affected by AP2 TFs, which is the target gene of miR172. *SlAP2a* is a negative regulator of tomato ripening [67], AP2a silences fruit ripening by producing more ethylene than WT, resulting in an early ripening phenomenon. Thus, miR172 is also a potentially important miRNA for fruit ripening [65]. ERF AP2 and B3 domain-containing transcription repressor was up-regulated in MT groups at S3, which is likely regulated by miR159a-5p.

Members of NAC family are involved in the regulation of ripening-associated processes in fruits, such as citrus [68], apple [69] and strawberry [70,71]. In cucumber, 12 *NAC* genes were found to target 13 known miRNAs which were involved in fruit spine development [72]. RT-qPCR revealed that the expression of *NAC100* was changed in response to miR164h-5p in MT. Low expression of miR164h-5p in MT triggered *NAC100* expressed during juice sac development. The regulatory relationship of miR164 and *NAC100* was also identified in the high-throughput sequencing of strawberry [73]. Recently, Wang revealed that the kiwi fruit miR164-NAC pathway was linked to fruit ripening and senescence [66] and this regulatory pathway for miR164-NAC is present in various fruit including citrus plants.

## 4. Materials and Methods

### 4.1. Quantification of Sugars, Organic Acids and Hormones

*Citrus grandis* ‘Guanximiyou’ (WT) and early ripening mutant ‘Liuyuezaoyou’ (MT) were grown in a pomelo orchard in Pinghe County, Fujian Province. Fruits of WT and MT were harvested at 76, 90, 104 and 118 DAF and immediately brought to the laboratory of Fujian Agriculture and Forestry University. After peeling off the outer skin of the fruits, the juice sacs were sampled from three fruits of WT and MT, which were prepared for measurement of sugars and organic acids contents.

Fruits of WT and MT at the four developmental stages were also sampled for high-throughput sequencing. Again, high-throughput sequencing and hormones content were conducted under three independent biological replicates of samples.

The samples for hormone quantification were prepared according to the method described by Wu [74] with some modifications, 1 g of juice sacs from pomelo fruit was ground with liquid nitrogen and placed into 10 mL centrifuge tube, 2 mL extract (isopropanol:water:concentrated hydrochloric acid = 2:1:0.002) was quickly added into centrifuge tube, 500 mL the mixed standard solution (50 ng/mL) was added and vortexed, then 4 mL dichloromethane was added and shaken over 200 r/min for 45 min. The supernatant was centrifuged at 4 °C at 10,000 rpm for 5 min and the supernatant was transferred to a new 10 mL centrifuge tube and dried with a nitrogen evaporator. The samples were redissolved in 800 mL of methanol. Finally, the solution was filtered through 0.22 mm nylon filter into a 2 mL brown chromatographic bottle for phytohormone testing.

The chromatographic conditions were Waters BEH-C18 column (2.1 mm × 100 mm, 1.7 μm) with water (HPLC grade containing 5 mmol/L ammonium acetate aqueous solution) and acetonitrile (HPLC grade containing) as mobile phase solvents A and B. The following gradient elution: 0–1 min, 2% acetonitrile; 1–2 min, 30% acetonitrile; 2–5 min, 95% acetonitrile; and 5–8 min, 2% acetonitrile. The flow rate was 0.3 mL/min, the column temperature was 30 °C and the injection volume was 10 μL. The mass spectrometry conditions were electrospray ion source (ESI), negative ion scanning, multi-reaction monitoring, (MRM), ion source temperature 150 °C. The data of mass spectrometry were collected and analyzed by Waters Masslynx 4.1 software (Waters, Milford, MA, USA). The experimental data were the average values of three replicates, which were expressed as the average value ± standard deviation (S.D.).

Organic acids were extracted according to the method described by Carballo [75] with some modifications, 2 g juice cells were ground to homogenate with 2 mL ddH_2_O and appropriate amount of quartz sand. The homogenate was transferred to a 10 mL centrifuge tubes. The supernatant was placed in a preheated water bath at 55 °C for 15 min, then centrifuged at 4000× *g* for 10 min), the supernatant was transferred into a 25 mL volumetric flask, the precipitate was extracted twice with 5 mL ddH_2_O, extracting solution was diluted to 25 mL, then 2 mL extract was diluted 50 times again, the solution was filtered with 0.22 mm water-based filter membrane.

ACQUITY-UPLC-MS (Agilent 1260 Infinity, Agilent 6410 triple Quadrupole Mass Spectrometer, Santa Clara, CA, USA) was used to determine the content of organic acids using ACQUITY-UPLC HSS T3 column (Waters, Milford, MA, USA). Water (HPLC grade containing 0.1% formic acid, *v*/*v*) and acetonitrile (HPLC grade containing 0.1% formic acid, *v*/*v*) were used for mobile phase solvents A and B, isocratic hold, flow rate: 0.2 mL/min; column temperature: 35 °C; injection volume: 5 mL. MassWork analysis software was used to calculate the content of soluble sugars by comparison of standard solution.

For soluble sugars extractions, 2 g juice cells were ground to homogenate with 2 mL ddH_2_O and appropriate amount of quartz sand, the homogenate was transferred to a 10 mL centrifuge tube. The supernatant was placed in a preheated water bath at 75 °C for 30 min, then centrifuged (10,000× *g*, 10 min), the supernatant was transferred into a 25 mL volumetric flask, the precipitate was extracted twice with 5 mL ddH_2_O, extracting solution was diluted to 25 mL, then 2 mL extract was diluted 5 times again, 1 mL the solution was filtered with 0.22 mm water-based filter membrane. The filtered solution was used for detection in UPLC-ELSD.

Ultraperformance liquid chromatography (ACQUITY UPLC (Waters, Milford, MA, USA) was used to determine the content of soluble sugars using WATERS ACQUITY UPLC BEH Amide column (100 × 2.1 mm, 0.5 mm). Acetonitrile (HPLC grade) and water (HPLC grade containing 0.1% *v*/*v* ammonia) and were used for mobile phase solvents A and B, A:B = 78%:22%, isocratic hold, flow rate: 0.2 mL/min; column temperature: 35 °C: injection volume: 2 mL. Other conditions were detection: ELSD: drift tube temperature: 40 °C, N_2_ flow rate = 1.5 L/min; gain: 1. WATERS EMPOVER3 software (Milford, MA, USA) was used to calculate the content of soluble sugars by comparison of standard solution.

Phytohormone, sugar and organic acid data were statistically analyzed using the SPSS software platform (25.0) (IBM Corporation, Somers, NY, USA). The mean difference for a specific compound between WT and MT at a particular stage were separated by *t*-test at *p* < 0.05 and *p* < 0.01 levels.

### 4.2. RNA Isolation, Small RNA Library Construction and High-Throughput Sequencing

Total RNAs of WT and MT samples were extracted from three biological replicates using the Trizol reagent kit (Invitrogen, Carlsbad, CA, USA) and then treated with DNase I (Fermentas, Carlsbad, CA, USA) by following the manufacturer’s instructions. The quantity and quality of prepared RNA were detected by Nanodrop, Qubit^TM^ RNA XR Assay Kit in Qubit2.0 and RNA 6000 Nano Kit in Agilent 2100 (Agilent Technologies Co., Ltd., Palo Alto, CA, USA). NEBNext Multiplex Small RNA Library Prep Set for Illumina (New England Biolabs, Ipswich, MA, USA) was used to construct the RNA library. Briefly, 3′SR adaptor and nuclease-free water was mixed with RNA, the mixture was denatured by a preheated thermal cycler for 2 min at 70 °C. The product was mixed with Ligation Enzyme Mix and Ligation Reaction Buffer (2X) at 25 °C for 1 h ligation reaction. Short fragment small RNAs with a length ranging from 18 to 40 nt were separated and purified from the total RNAs by polyacrylamide gel electrophoresis (PAGE) after adaptor ligation. The purified RNAs were used for reverse transcription and subsequent PCR. Deep sequencing was performed using an Illumina HiSeq 2000 platform at Beijing Biomarker Technologies (Beijing, China).

The mRNAs were purified from the total RNA to generate the cDNA library according to NEBNext Ultra RNA Library Prep Kit for Illumina. First, mRNAs were purified from total RNA using Poly-T Oligo magnetic beads (Catalog No. S1419S, New England Biolabs, NEB, Lpswich, MA, USA). The first cDNA was synthesized using random hexamer primers and M-MLV reverse transcriptase and the second cDNA was synthesized by DNA polymerase Ⅰ and RNase H. The remaining protruding ends are converted to flat ends by exonuclease/polymerase activity. After adenylation of the 3′ end of the DNA fragment, Neb next Adaptor was attached to the hairpin ring structure, ready for hybridization. To screen for cDNA fragments with a preference length of 240 bp, the library fragments were purified using the AMPure XP system (Beckman-Coulter, Beverly, MA, USA). The cDNA was ligated at 37 °C for 15 min and then kept at 95 °C for 5 min. PCR amplification was performed using Phusion High-Fidelity DNA Polymerase, universal PCR primer and index (X) primer. Finally, PCR product purification (AMPure XP system) and library detection were performed and library detection was performed on the Agilent BioAnalyzer 2100 system with DNA 1000 Kit. Truseq PE Cluster Kit v3-CBOT-HS (Illumina) was used to perform the clustering of index coded samples on the CBOT Cluster generation system. After clustering, the library was prepared for sequencing on Illumina HiSeq 2000 platform and 125/150 bp paired-end reads were generated.

To obtain the clean reads, low-quality reads and trimming adapter sequences were removed by FastQC tools (https://www.bioinformatics.babraham.ac.uk/projects/fastqc/ accessed on 24 August 2021) High-quality clean reads were assembled to produce non-redundant transcripts using the program Trinity (http://trinityrnaseq.github.io/). The mRNAs were identified based on the annotation information of the *Citrus grandis* genome (http://citrus.hzau.edu.cn/orange/ accessed on 24 August 2021). miRNAs were predicted via the miRDeep2 software aligning with known miRNAs present in the miRbase [76]. Further, DESeq R package was used to identify significant differential miRNA between the libraries [77]. The relationship between target genes and miRNAs were predicted by an online program psRNATarget (http://plantgrn.noble.org/psRNATarget/ accessed on 24 August 2021) [78]. To study the function of differentially expressed genes and the target genes of miRNAs, Clusters of Orthologous Groups of proteins (COG) and KEGG (http://www.genome.jp/kegg/ accessed on 24 August 2021) enrichment analyses were performed and verified.

### 4.3. Validation of mRNA and miRNAs Expressions by Quantitative Real-Time PCR Analysis

To quantify the identified miRNAs and mRNAs, poly(A) extension RT-qPCR was performed using a protocol with minor modifications [79]. All the RT-qPCR templates were generated from 3 µg total RNA isolated from the WT and MT juice sacs at 76, 90, 104 and 118 DAF. cDNAs for miRNA and mRNA quantification were synthesized using One Step PrimeScript miRNA cDNA Synthesis Kit (Takara, Beijing, China) and M-MLV cDNA Synthesis Kit (Promega, Madison, WI, USA). Each amplification was performed in a 20-μL reaction system containing 10 mL of qPCR SYBR^®^ Green Master Mix (Takara), 0.8 mL of each specific primer pair, 1.0 μL of 10-fold diluted cDNAs and 7.4 mL of ddH_2_O. RT-qPCR was performed on the Bio-Rad CFX96 TouchTM RealTime PCR Detection System using a standard SYBR Green PCR Kit (Bio-Rad, Hercules, CA, USA). Reference genes *EF1a* and *Actin* were used to normalize data. Relative expression levels of each mRNA and miRNA were determined using the 2−^ΔΔ^CT method [80]. All reactions were performed in independent biological and technical triplicates. All primers used in this test were listed in Additional file 3: Table S2. The relationship between a miRNA and its targeted mRNA in WT and MT was analyzed, respectively based on Pearson’s correlation analysis and coefficients were presented.

## 5. Conclusions

The present study analyzed temporal changes in key phytohormones, sugar compounds and organic acids in an early maturing mutant pomelo and its parental type and investigated the occurrence of miRNAs and their interactions with targeted mRNAs in regulation of fruit ripening. The early maturing fruit mutant was accompanied with significant changes in ABA, IAA, sugar, and organic acids at different ripening stages. More importantly, miRNAs were found to be heavily involved in the early ripening process. A total of 747 known miRNAs were identified and 99 novel miRNAs were predicted. The expression of miRNA negatively regulated the expression of targeted mRNAs involved in primary metabolism, cell-wall degradation, phytohormones and transcription factors during the fruit ripening process. Some noticeable targets include auxin-responsive protein IAA9, sucrose synthase 3, V-type proton ATPase, NCED1, PL1/5 and NAC100. Compared to the WT, the early maturation appears to be largely attributed to more active actions of those miRNAs to respective targets. Further research is needed to verify this proposition and elucidate the underlying mechanisms.

## Figures and Tables

**Table 1:** Summary of the mRNA sequence analyses.

Samples	Clean Reads	Clean Bases	Mapped Reads	% ≥ Q30
S1-MT-1	26,640,915	5,328,183,000	50,919,858 (95.57%)	91.00%
S1-MT-2	27,829,729	5,565,945,800	53,227,972 (95.63%)	90.95%
S1-MT-3	25,677,502	5,135,500,400	49,003,676 (95.42%)	89.63%
S1-WT-1	27,212,122	5,442,424,400	51,501,260 (94.63%)	90.53%
S1-WT-2	27,138,832	5,427,766,400	51,825,236 (95.48%)	89.11%
S1-WT-3	27,311,316	5,462,263,200	52,304,961 (95.76%)	89.58%
S2-MT-1	27,869,332	5,573,866,400	53,346,900 (95.71%)	91.20%
S2-MT-2	26,821,833	5,364,366,600	50,950,626 (94.98%)	89.47%
S2-MT-3	26,817,092	5,363,418,400	51,321,446 (95.69%)	89.74%
S2-WT-1	25,982,460	5,196,492,000	49,078,023 (94.44%)	90.21%
S2-WT-2	27,193,142	5,438,628,400	51,895,992 (95.42%)	89.67%
S2-WT-3	28,451,231	5,690,246,200	53,949,964 (94.81%)	89.58%
S3-MT-1	26,636,948	5,327,389,600	50,830,277 (95.41%)	90.90%
S3-MT-2	27,670,745	5,534,149,000	52,826,553 (95.46%)	90.62%
S3-MT-3	26,836,621	5,367,324,200	51,491,386 (95.93%)	89.56%
S3-WT-1	27,180,721	5,436,144,200	51,593,053 (94.91%)	90.20%
S3-WT-2	27,210,453	5,442,090,600	51,631,745 (94.87%)	89.43%
S3-WT-3	27,708,186	5,541,637,200	52,587,039 (94.89%)	89.62%
S4-MT-1	25,947,281	5,189,456,200	49,700,389 (95.77%)	89.98%
S4-MT-2	26,559,409	5,311,881,800	50,536,470 (95.14%)	89.27%
S4-MT-3	26,864,690	5,372,938,000	51,571,664 (95.98%)	89.58%
S4-WT-1	27,584,243	5,516,848,600	52,631,279 (95.40%)	89.89%
S4-WT-2	26,102,323	5,220,464,600	49,866,893 (95.52%)	89.54%
S4-WT-3	27,546,842	5,509,368,400	52,424,202 (95.15%)	90.24%

**Table 2:** Identification of known and novel miRNAs.

BMK-ID	Known-miRNAs	Novel-miRNAs	Total
S1-MT	461	99	560
S1-WT	482	99	581
S2-MT	487	99	586
S2-WT	442	99	541
S3-MT	472	99	571
S3-WT	437	99	536
S4-MT	452	99	551
S4-WT	423	99	522
Total	747	99	846

## Data Availability

The eight small RNA sequences were submitted to NCBI BioProject with an accession number PRJNA755343 (https://www.ncbi.nlm.nih.gov/bioproject/PRJNA755343 accessed on 27 August 2021).

## Supplementary Materials

The following are available online at www.mdpi.com/article/xxx/s1. Additional file 1: Table S1. Differentially expressed microRNA targets in juice sacs of WT and MT fruits. Additional file 2: Figure S1. miRNA target proteins analyzed by COG function classification of consensus sequences annotation. Additional file 3: Table S2: Primers.
