# Supplementary material for: MicroRNAs and Transcripts Associated with an Early Ripening Mutant of Pomelo (Citrus grandis Osbeck)
Source: Int J Mol Sci. 2021 Aug 28;22(17):9348. doi: 10.3390/ijms22179348 (PMC8431688; doi:10.3390/ijms22179348)
Supplement: Supplementary file 1 [file ijms-22-09348-s001.zip › ijms-1299429-Additional file 1. Table S1.pdf]

International Journal of *Molecular Sciences*

MDPI

Additional file 1. Table S1. Differentially expressed microRNA targets in juice sacs of WT and MT fruits.

MiRNA	Mature miRNA sequence	Target ID	Target annotation	Target gene Expression pattern (MT/WT)	Function Description
hbr-miR6483	UAUUGUAGAAAUUUUCAGGAUC	Cg1g013430	ATPase 10	S1 down	H+-transporting ATPase
gma-miR1521b	GACUGUCACGUGUCAUAAUCAUA	Cg5g035630	bHLH-MYC and R2R3-MYB transcription factors	S1 down	Transcription
ath-miR395a	GUGAAGUGUUUGGAGGAACUC	Cg3g024040	Gibberellin-regulated protein 1	S1 up	Response to stimulus, developmental process
csi-miR530a-5p	UGCAUUUGCACCUGCACCUUG	Cg6g002040	V-type proton ATPase subunit G	S1 up	Oxidative phosphorylation, hydrolase activity, acting on acid anhydrides
ath-miR165a-3p	UCGGACCAGGCUUCAUCCCC	Cg3g013510	Auxin-responsive protein IAA9	S1 up	Plant hormone signal transduction
csi-miR482d-5p	CGGCUCUGAUACCAGUUGAUG	Cg5g037720	Sucrose synthase 3	S1 up	Cell wall/membrane/envelope biogenesis, Starch and sucrose metabolism
mtr-miR5293	GAUGAAGAAGUGGAAGGAAGAAGA	Cg5g039890	Myb-like DNA-binding domain	S1 up	MYB-related transcription factor LHY, Posttranslational modification
ptc-miR395a	CUGAAGGGUUUGGAGGAACUC	Cg2g028850	Purple acid phosphatase 8	S1 up	Acid phosphatase activity, Posttranslational modification, protein turnover, chaperones
ath-miR825	UUCUCAAGAAGGUGCAUGAAC	Cg3g018840	GIGANTEA	S1 up	Circadian rhythm
cme-miR858	UCUCGUUGUCUGUUCGACCUU	Cg7g003710	Chalcone-flavonone isomerase 3	S1/S2 down	Flavonoid biosynthetic
ath-miR167d	UGAA-GCUGCCAGCAUGAUCU	Cg6g006940	E3 ubiquitin-protein ligase	S1/S2 down	Metal ion binding, posttranslational modification, protein turnover, chaperones
csi-miR391-5p	UGCAGGUGAGAUGAUACCGUCA	Cg3g018510	Abscisic stress-ripening protein 2-like	S1/S2 down S3 up	Response to stimulus
novel_miR_32	AUAAGAGUUUGUGACUAUAUCAUU	Cg7g016790	MADS-box protein FLOWERING LOCUS C	S1/S2 up	Transcription factor activity, sequence-specific DNA binding
aly-miR390b-3p	CGCUAUCCAUCCUGAGUUCCA	Cg8g007130	Isoflavone reductase	S1/S2/S3 up	Secondary metabolites biosynthesis
mes-miR535a	UGACAACGAGAGAGA-GCACGU	Cg3g015750	Eukaryotic translation initiation factor 5-2	S1/S2/S3/S4 up	Translation, ribosomal structure and biogenesis

ath-miR1888a	UAAGUUAAGAUUUGUGAAGAA	Cg9g024430	glutamine amidotransferase YLR126C	S1/S2/S3/S4 up	Nucleotide transport and metabolism
ath-miR171c-5p	AGAUAUUGGUG-CGGUUCAAUC	Cg1g013440	Leucine-rich repeat receptor-like protein kinase	S1/S2/S4 down	Signal transduction mechanisms, transcription
ath-miR5021	UGAGAAGAAGAAGAAGAAAA	Cg5g008630	Acid beta-fructofuranosidase 38 kDa subunit	S1/S2/S4 up	Galactose metabolism, Starch and sucrose metabolism
bdi-miR9480a	UAUGUGAGGGUGGUAA-CUGAA	Cg2g009320	xyloglucan endotransglucosylase/hydrolase protein 5	S1/S2/S4 up	Carbohydrate transport and metabolism
ath-miR394a	UUGGCAUUCUGUCCACCUCC	Cg6g024860	Mini zinc finger protein 2	S2 down	ZF-HD protein
novel_miR_15	CACCAGUCGUUGGAUCCACUCAA	Cg6g017010	Rhodanese-like domain-containing protein 6	S2 down	Amino acid transport and metabolism
ath-miR5654-5p	AUAAAUCCCAACAUCUUCCA	Cg7g003370	WEB family protein	S2 down	Weak chloroplast movement
csi-miR482e-5p	GGUCAUGGGAGGAUUGGCGA	Cg2g020420	Nitrile-specifier protein 5	S2 down	Response to zinc ion , galactose oxidase
lus-miR159b	UUUGGAUUGAAGGGAGCUCUC	Cg8g002360	Beta-glucosidase 11	S2 down	Carbohydrate transport and metabolism
ath-miR159a	UUUGGAUUGAAGGGAGCUCUA	Cg2g042640	Triose phosphate/phosphate translocator TPT	S2 down	Transporter activity
csi-miR160c-3p	GCGUGCGAGGAGCCAUGCAUG	Cg4g008660	UDP-glycosyltransferase	S2 down	Transferase activity, transferring glycosyl groups
aau-miR168	AUUCAGUUGAUGCAAGGCGGGAUC	Cg1g004930	Beta-galactosidase 3	S2 down	Carbohydrate transport and metabolism
aly-miR172e-3p	GAAUCUUGAUGAUGCUGCAU	Cg6g002830	Floral homeotic protein APETALA 2	S2 down	Transcription, oxidoreductase activity
ath-miR399b	UGCCAAAGGAGAGUUGCCCUG	Cg9g010980	Pectate lyase 1	S2 up	Carbohydrate transport and metabolism
aly-miR845b-5p	AGUGGAAGUAGCAAGGGGAAGC	Cg9g002720	Late embryogenesis abundant protein Lea5	S2 up	Response to stimulus
csi-miR3951b-5p	UAGAUAAAGAUGAGAGAAAA	Cg5g006150	Pectate lyase 5	S2 up	Pentose and glucuronate interconversions, carbohydrate transport and metabolism
ath-miR169h	AUUCAGUUGAUGCAAGGCGGGAUC	Cg5g039580	Papain family cysteine protease	S2 up	Posttranslational modification, protein turnover, chaperones
csi-miR482c-5p	UUUUGCUCAAGACCGCGCAAC	Cg9g003540	Inactive beta-amylase 9	S2 up	Glycosyl hydrolase family 14
ath-miR1886.2	UGAGAUGAAAUCUUUGAUUGG	Cg6g023440	Gibberellin-regulated protein 14	S2 up	Response to gibberellin
csi-miR12107-3p	UCAUUCGCGCUCUCAUCAUUA	Cg5g013480	L-cysteine desulfhydrase	S2 up	Amino acid transport and metabolism

csi-miR3951b-5p	CAGGUAAAGAUGAGAGAAAAA	Cg7g010980	Transcription factor bHLH137	S2 up	Protein dimerization activity
csi-miR3952-3p	UGAAGGGCCUUUCUAGAGCAC	Cg5g009980	Bifunctional UDP-glucose 4-epimerase and UDP-xylose 4-epimerase 1	S2 up	Cell wall/membrane/envelope biogenesis, galactose metabolism
aqc-miR171f	UAAUUGAGCCGUGCCAAUAUC	Cg5g001400	Endoglucanase 6	S2 up	Carbohydrate metabolic process, cellulase activity
csi-miR9560-5p	ACAGGAGGUGGAACAAAUAUGAAA	Cg7g001220	Auxin transporter-like protein 1	S2 up	Plant hormone signal transduction ,Amino acid transport and metabolism
bdi-miR7753-3p	UGAGCAAGGGAGAAGACAUGG	Cg5g036390	Gibberellin receptor GID1B	S2 up	Lipid transport and metabolism, hydrolase activity, plant hormone signal transduction
mtr-miR156b-3p	UGCUCACUCUCUAUCUGUCA	Cg5g006850	SNF1-related protein kinase regulatory subunit gamma-1	S2 up	Kinase activity ,phosphorylation
bdi-miR7712-5p	UAGAGCUCUGAAGUUACCACCCAC	Cg3g016650	IAA-amino acid hydrolase ILR1-like 6	S2 up	Auxin metabolic process, IAA-amino acid conjugate hydrolase activity
ath-miR164a	UGGAGAAGCAGGGCACGUGCA	Cg6g025130	NAC domain-containing protein 100	S2 up	DNA binding ,regulation of transcription
gma-miR10195	UAUGAUUUUGUGGAUCAAAGGA	Cg9g000960	Cell wall / vacuolar inhibitor of fructosidase 1	S2 up	Plant invertase/pectin methylesterase inhibitor
ath-miR164a	UGGAGAAGCAGGGCACGUGCA	Cg2g044950	9-cis-epoxycarotenoid dioxygenase NCED1	S2 up S3 down	Secondary metabolites biosynthesis, transport and catabolism
aly-miR861-5p	GUUUGGAGAAAUAUGCAUCAU	Cg5g010450	Galactoside 2-alpha-L-fucosyltransferase	S2/ S4 down	Xyloglucan fucosyltransferase
gma-miR4995	AGGCAGUGGCUUGGUUAAGGG	Cg3g021950	Dof zinc finger protein DOF1.6	S2/ S4 up	Dof domain, zinc finger
nta-miR477a	UUUCGGUGUCGGUGAAUUGCC	Cg2g045100	Peroxidase 42	S2/S3 down	Phenylpropanoid biosynthesis, carbohydrate transport and metabolism
gma-miR4372b	UAAUAAAAUCGUGACAUGUAAC	Cg6g010490	Non-specific lipid-transfer protein	S2/S3 down	Protease inhibitor/seed storage
csi-miR536-5p	CGCACCCCAGCGUGGAACCAUC	Cg5g014880	Methyltransferase PMT14	S2/S3 down	Methyltransferase activity
novel_miR_11	UAGCCAUUGUUGUUGUUGGAA	Cg5g012600	Cytochrome P450 82C4	S2/S3 down	Secondary metabolites biosynthesis, transport and catabolism Cytochrome P450
csi-miR403b-5p	AGUUUGUGCGUGAAUCUAACC	Cg2g009540	Chalcone synthase 2	S2/S3 down	Secondary metabolites biosynthesis, transport and catabolism

aly-miR157d-3p	GCUCUCUAUGCUUCUGUCAUC	Cg2g001370	Peroxidase 15	S2/S3 down	Peroxidase activity
ath-miR5653	UGGGUUGAGUU-GAGUUGAGUUGGC	Cg5g014390	Fasciclin-like arabinogalactan protein 17	S2/S3 down	Cell wall/membrane/envelope biogenesis
aly-miR165a-5p	GAAUGUUGUCUGGAUCGAGG	Cg6g009720	Ferritin-3	S2/S3 down	Inorganic ion transport and metabolism
cme-miR399g	AGGGCUUCUCUCCAUUGGCAGG	Cg5g029360	Caffeic acid 3-O-methyltransferase	S2/S3 down	Secondary metabolites biosynthesis
aly-miR845a-3p	CGGCUCUGAUACCAGUUGAUG	Cg7g000350	UDP-glycosyltransferase 76F1	S2/S3 down	Carbohydrate transport and metabolism, energy production and conversion
novel_miR_11	AAGC UUUGUCAUUUGUGGUA	Cg4g004810	Vacuolar-processing enzyme	S2/S3 up	Posttranslational modification, protein turnover, chaperones
csi-miR156e-5p	GUGACAGAAGAUAGAGAGCGC	Cg8g022140	Mediator of RNA polymerase II transcription subunit	S2/S3 up	Posttranslational modification, protein turnover, chaperones
csi-miR477e-3p	UGAGGUUCUUGGGGAGAGUAG	Cg4g017510	Thiol protease aleurain	S2/S3 up	Proteolysis, posttranslational modification, protein turnover, chaperones
bdi-miR7712-5p	UAGAGCUCUGAAGUUACCACCCAC	Cg3g010060	Pectin acetylesterase 8	S2/S3 up	Carboxylic ester hydrolase activity wall/membrane/envelope biogenesis
bna-miR156a	UGACAGAAGAGAGUGAGCAC	Cg5g030400	Cytochrome P450 83B1	S2/S3 up	Secondary metabolites biosynthesis, transport and catabolism
ath-miR167d	UGAAGCUGCCAGCAUGAUCUGG	Cg2g008540	Protein trichome birefringence-like 39	S2/S3 up	
novel_miR_73	CAUCCUUGAAAUCUGAUUGG	Cg9g003780	Sugar transporter ERD6-like 16	S2/S3 up	Carbohydrate transport and metabolism
csi-miR12106-3p	UGGGGCAGCUGUCCUAAACGG	Cg1g003690	BES1/BZR1 homolog protein 2	S2/S3 up	Plant hormone signal transduction
aly-miR395i	CUGAAGUGUUUGGAGGAACUC	Cg3g024030	Snakin-2	S2/S3 up	Polysaccharide biosynthetic process
mtr-miR166b	UCGGACCAGGCUUCAUUCCUA	Cg1g016140	ABC-transporter	S2/S3 up	Secondary metabolites biosynthesis, transport and catabolism
gma-miR159a-5p	GAGCUCCUUGAAGUCCAAUUG	Cg2g018720	Cellulose synthase-like protein B4	S2/S3 up	Cell wall/membrane/envelope biogenesis
ath-miR165a-3p	UCGGACCAGGCUUCAUCCCC	Cg2g041500	Ctenidin-3-like	S2/S3/S4 down	
ath-miR156i	UGACAGAAGAGAGAGAGCAG	Cg9g004610	Copper transport protein	S2/S3/S4 down	Metal ion transport

novel_miR_63	UAAAAUGCGGAUUUGUUGUAUAC	Cg5g040600	Ethylene-responsive transcription factor ERF023	S2/S3/S4 down	Transcription
novel_miR_20	UAGGAUGUAGAAGAGCAUAA	Cg8g018270	Glucomannan 4-beta-mannosyltransferase 2	S2/S3/S4 down	Cell wall/membrane/envelope biogenesis
ath-miR395a	CUGAAGUGUUUGGGGGAACUC	Cg6g020720	Pollen proteins Ole e I like	S2/S3/S4 up	
osa-miR5505	GAGGAUUCGGUAUUGAUCGCUA	Cg2g038980	Tetrahydrocannabinolic acid synthase	S2/S4 down	Flavin adenine dinucleotide binding
mtr-miR2595	GGUGUUCUCAGGUCGCCCCUG	Cg5g037690	Pectinesterase 40	S2/S4 down	Pentose and glucuronate interconversions, Starch and sucrose metabolism
novel_miR_63	UAAAAUGCG-GAUUUGUUGUAUAC	Cg1g006400	LOB domain-containing protein	S2/S4 down	Transcription
ath-miR8175	GAUCCCCGGCAACGGCGCCA	Cg5g010350	Expansin-A1	S2/S4 up	Plant-type cell wall organization
ata-miR5200-5p	AAGCCUUAGUG-AAUAUCUACA	CgUng002730	UDP-glycosyltransferase	S2/S4 up	Carbohydrate transport and metabolism, energy production
ath-miR167a-5p	UGAAGCUGCCAGCAUGAUCU	Cg1g002060	Naringenin,2-oxoglutarate 3-dioxygenase	S3 down	Oxidoreductase activity
ath-miR390a-5p	AAGCUCAGGAGGGAUAGCGCC	Cg4g003490	Dehydration-responsive protein RD22	S3 down	Response to stimulus
mdm-miR10999a	GGGCGUGAUAUUCACACACCU	Cg6g010480	Non-specific lipid-transfer protein 1	S3 down	Lipid transport
csi-miR477c-3p	GAAGUCCUUGGGGUUGAGUGA	Cg3g021110	WUSCHEL-related homeobox 4	S3 down	Transcription factor activity, sequence-specific DNA binding
ath-miR162a-5p	GGAGGCAGCGGUUCAUCGAUC	Cg1g029080	Cellulase (glycosyl hydrolase family 5)	S3 down	Carbohydrate transport and metabolism
ath-miR164a	UGGAGAAGCAGGGCACGUGCA	Cg9g009400	Peroxygenase 1	S3 down	Secondary metabolites biosynthesis
ath-miR396a-5p	UUCCACAGCUUUCUUGAACUG	Cg6g017060	Magnesium-protoporphyrin IX monomethyl ester	S3 down	DNA binding, rRNA processing , unsaturated fatty acid biosynthetic process
ath-miR156i	UGACAGAAGAGAGAGAGCAG	Cg6g016460	leucine-rich repeat receptor-like protein kinase	S3 down	Signal transduction , Transcription
aly-miR827-3p	UUAGAUGACCAUCAACAAACG	Cg5g011730	MADS-box protein CMB1	S3 down	DNA binding, transcription factor activity
csi-miR12105-5p	GUUCCCAUGCCACCCAUUUCUA	Cg8g020730	Elongation factor 1-alpha	S3 up	Translation, ribosomal structure and biogenesis
ath-miR172c	GAAUCUUGAUGAUGCUGCAU	Cg5g037520	Indole-3-acetic acid-induced protein	S3 up	Response to stimulus

osa-miR2121a	UGAACUCUCCCCCUCAACGGCU	Cg6g003110	NEDD8-like protein RUB2	S3 up	Posttranslational modification, protein turnover, chaperones
zma-miR2275d-3p	UUUGUUUUCCUCUAAUAUCUCA	Cg6g025030	Calcium-binding protein CML45	S3 up	Signal transduction mechanisms, cytoskeleton, cell cycle control, cell division pair
csi-miR3948	UGGAGUGGGAGUGGGAGUAGGGUG	Cg5g037790	Ethylene-responsive transcription factor RAP2-3	S3 up	DNA binding ,transcription factor activity
csi-miR857	UUUUGAAUGUUGAAUGGUGGCUAU	Cg5g040920	WRKY transcription factor 53	S3 up	Transcription
ath-miR171a-3p	UGAUUGAGCCGCGCCAAUAUC	Cg5g001540	WRKY transcription factor 41	S3 up	Transcription
csi-miR3948	UGGAGUGGGAGUGGGAGUAGGGUG	Cg4g019180	NOL1/NOP2/sun family	S3 up	Translation, ribosomal structure and biogenesis
tae-miR1134	CAACAACAACAAGAAGAAGAAGAU	Cg1g013340	RING-H2 finger protein ATL60	S3 up	Posttranslational modification, protein turnover, chaperones
aly-miR159a-5p	GAGCUCCUUGAAGUUCAAACG	Cg7g014820	AP2/ERF and B3 domain-containing transcription repressor	S3 up	DNA binding, transcription factor activity
miR156b-3	GCUCACUCUCUAUCUGUCAGC	Cg8g018250	Histone deacetylase HDT1	S3/S4 up	Chromatin structure and dynamics, DNA mediated transformation
osa-miR5491	CCCGGCAUUCAUGUUUUCCU	Cg4g019480	Galactinol--sucrose galactosyltransferase 6	S4 down	Galactose metabolism
csi-miR408-5p	ACGGGGAACAGGCAGAGCAUG	Cg9g008300	Methyltransferase PMT8	S4 down	Secondary metabolites biosynthesis
ath-miR5658	AUGAUGAUGAUGAUGAUGAAA	Cg1g010660	Linoleate 13S-lipoxygenase 3-1	S4 down	Iron ion binding ,alpha-Linolenic acid metabolism
bdi-miR169h-3p	GGCAGUCACCUUGGCUAGC	Cg7g021980	UDP-glucose 6-dehydrogenase 3 GN	S4 down	Cell wall/membrane/envelope biogenesis, oxidation-reduction process
ath-miR1888a	UAAGUUAAGAUUUG-UGAAGAA	Cg3g016320	Ethylene-responsive transcription factor ERF012	S4 down	Transcription
aly-miR165a-3p	UCGGACCAGGCUUCAUCCCC	Cg9g019270	Heat stress transcription factor C-1	S4 down	Transcription factor activity, response to stimulus
osa-miR159c	AUUGGAUUGAAGGGAGCUCCA	Cg2g020660	Zinc finger protein CONSTANS-LIKE 9	S4 down	Zinc ion binding
ath-miR171c-5p	AGAUAUUGGUGCGGUUCAAUC	Cg3g021860	Mitogen-activated protein kinase kinase kinase YODA	S4 down	Signal transduction, transcription

mtr-miR5284h	GAGGGAUCAAAAGUGGAGGA-AUCU	Cg1g010830	TIFY 10A	S4 down	Plant hormone signal transduction
csi-miR9560-3p	AGAAGAGAGAGAGUACAGCCC	Cg5g002580	Aquaporin PIP1-2	S4 up	Response to water deprivation, carbohydrate transport and metabolism
sly-miR6024	GAGGAUUCGGUAUUGAUCGCUA	Cg2g046060	Tonoplast dicarboxylate transporter	S4 up	Inorganic ion transport and metabolism
