# Supplementary material for: MicroRNAs and Transcripts Associated with an Early Ripening Mutant of Pomelo (Citrus grandis Osbeck)
Source: Int J Mol Sci. 2021 Aug 28;22(17):9348. doi: 10.3390/ijms22179348 (PMC8431688; doi:10.3390/ijms22179348)
Supplement: Supplementary file 1 [file ijms-22-09348-s001.zip › ijms-1299429-Additional file 2_Figure S1.pdf]

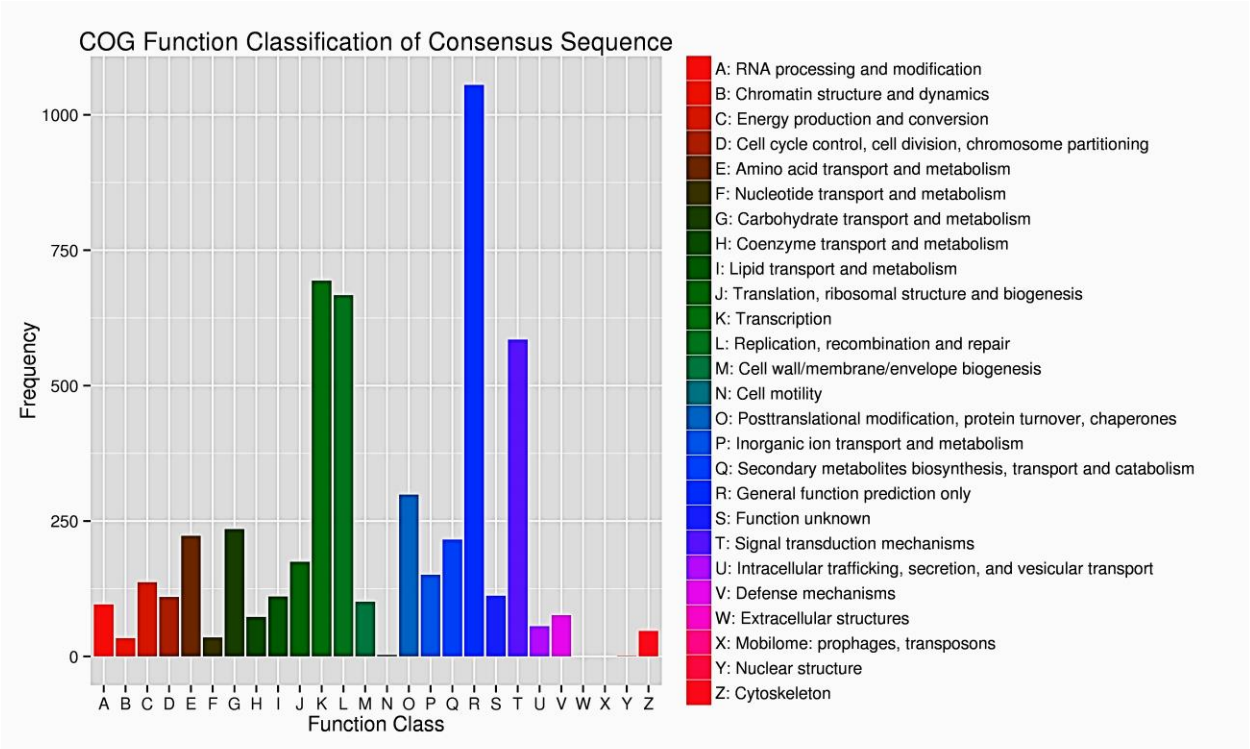

Additional file 2: Figure S1. miRNA target proteins analyzed by COG function classification of consensus sequences annotation.
